# A Very Bad Hair Day: Minoxidil Ingestion Causing Shock and Heart Failure

**DOI:** 10.7759/cureus.66039

**Published:** 2024-08-02

**Authors:** Sabin Tripathee, Alexander Benyovszky, Ruchi Devbhandari, Katherine Quiza, James Boris

**Affiliations:** 1 Internal Medicine, Jefferson Einstein Montgomery Hospital, East Norriton, USA

**Keywords:** midodrine, shock, heart failure, overdose, minoxidil

## Abstract

Minoxidil is a potent directly acting vasodilator previously used in treatment-resistant hypertension. It possesses several serious side effects including fluid retention, worsening of heart failure, reflex tachycardia, angina, myocardial infarction, pericardial effusion, and hypotension. It is currently reserved for treating alopecia and readily available over the counter as a topical formulation. Intentional/accidental ingestion of topical minoxidil can cause refractory circulatory shock requiring aggressive hydration and vasopressor support. We present a case of a young female with unintentional ingestion of minoxidil leading to severe circulatory shock and acute pulmonary edema. Minoxidil, a common hair loss treatment, is highly dangerous if ingested. Immediate identification and treatment are crucial, involving fluid resuscitation and vasopressors for severe circulatory shock. Midodrine, an alpha-adrenergic agonist, can reduce ICU stay by shortening IV vasopressor usage.

## Introduction

Minoxidil is a potent, directly acting vasodilator previously used in treatment-resistant hypertension. Its vasodilatory effect is mediated via ATP-sensitive potassium channel opening activity, which causes hyperpolarization of the cells, resulting in vascular smooth muscle relaxation. Its use in hypertension is limited by several serious side effects, including fluid retention causing or worsening heart failure, severe reflex tachycardia leading to angina or even myocardial infarction, pericardial effusion/tamponade, hypotension, and shock [1].

Minoxidil is currently reserved for treating alopecia due to various causes. It is easily available over the counter as a topical formulation in strengths of either 2% or 5% in a 60 mL bottle. Intentional or accidental ingestion of topical minoxidil can cause severe hypotension and refractory circulatory shock. Treatment is supportive with aggressive hydration, vasopressor support, and possibly mechanical ventilation and diuretics for fluid overload, due to the absence of a specific antidote [2-6].

We describe a case of a 17-year-old female with accidental ingestion leading to severe circulatory shock, initially followed by the development of acute heart failure. This case was previously presented as a meeting abstract at the American College of Physicians Southeastern Regional Posters Day and Doctors Dilemma at Abington, Pennsylvania on October 10, 2023.

## Case presentation

A 17-year-old Spanish-speaking female with no significant past medical history presented to the emergency department for evaluation of chest pain and tachycardia following the accidental ingestion of 60 mL of 5% topical minoxidil (3000 mg). According to the patient, she started experiencing chest discomfort after drinking half a can of Monster. She then reported drinking an entire bottle of Rogaine, which her father used for hair loss treatment, mistaking it for an antacid. Upon arrival, the patient was in circulatory shock with a blood pressure of 87/42 mmHg and a heart rate of 152 bpm. Cardiovascular examination was normal, with normal S1 and S2, and without any murmurs, rubs, or gallops. Respiratory examination revealed normal bilateral vesicular breath sounds with no added sounds. Abdominal examination was unremarkable. Initial blood work showed lactic acidosis of 3.63 and mild transaminitis; otherwise, results were largely unremarkable. The initial chest X-ray was normal (Figure 2, left).

Following a toxicology evaluation that recommended conservative management, she was admitted to the ICU and initiated on vasopressor support with phenylephrine. The patient experienced worsening acidemia from lactic acidosis due to persistent hypotension, leading to an increased requirement for norepinephrine. She was started on a bicarbonate drip, which led to improvement in acidemia and a decrease in phenylephrine requirement from 250 mcg/min to 50 mcg/min. She was eventually weaned off phenylephrine and started on midodrine 5 mg TID.

**Figure 1:**
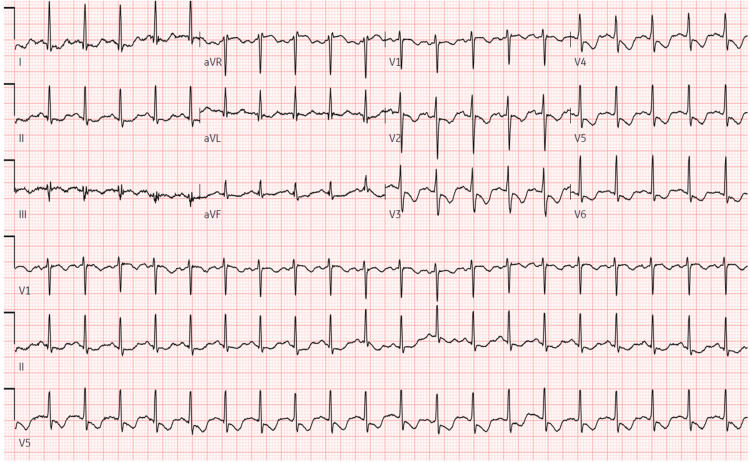
EKG showing anterolateral ST-segment depression and T-wave inversion reflecting subendocardial ischemia

The initial EKG showed ST-segment depression in V3-V6 with T-wave inversion in V1-V6 (Figure 1). Although the initial troponin was normal at 0.02, it continued to trend up and peaked at 0.63. The elevated troponin and initial EKG changes were thought to be secondary to Type II myocardial infarction from tachycardia and a high cardiac output state. EKG findings continued to improve with supportive management and resolution of tachycardia.

**Figure 2:**
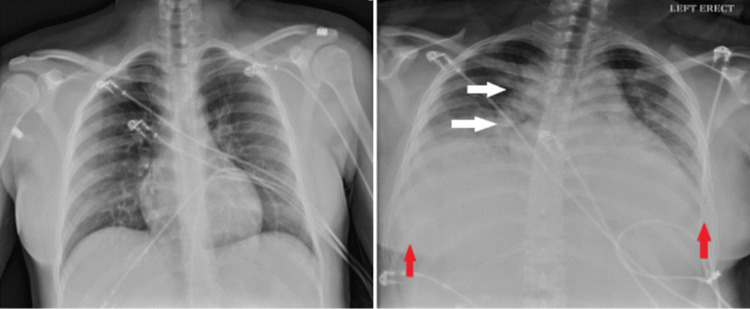
Normal chest X-ray on admission (left) and chest X-ray on day 2 (right) notable for pulmonary edema (white arrows) and bilateral pleural effusions (red arrows) with cardiomegaly

On the second day of admission, the patient began experiencing shortness of breath. A repeat chest X-ray showed diffuse pulmonary edema and small bilateral pleural effusions (Figure 2, right), likely secondary to fluid retention and possibly worsened by initial aggressive hydration. Elevated brain natriuretic peptide (BNP) levels suggested a possible component of tachycardia-induced high cardiac output heart failure. Transthoracic echocardiography showed a hyperdynamic left ventricle with a left ventricular ejection fraction (LVEF) of 70% and mild mitral and tricuspid regurgitation. Fluid overload improved rapidly with furosemide. Midodrine was continued to prevent hypotension in the setting of a recent profound circulatory shock.

On day four, we were able to wean her off midodrine, and furosemide was discontinued. She was downgraded to the telemetry unit and eventually discharged after a five-day hospital stay with no further complications or permanent organ damage.

## Discussion

Minoxidil overdose can cause a broad range of serious cardiovascular side effects, including severe circulatory shock due to its direct vasodilatory effect, fluid retention, pulmonary edema, tachycardia-induced acute heart failure, and subendocardial ischemia. These complications were all present in our patients, making the treatment process complex.

The circulatory shock from minoxidil is usually severe, requiring vasopressor support. Previous case reports have documented that circulatory shock secondary to minoxidil toxicity has been managed with dopamine alone or in combination with phenylephrine due to their predominant alpha-agonist action, which counteracts the vasodilatory effects of minoxidil. Phenylephrine was preferred over dopamine in our case due to the increased risk of pro-arrhythmias associated with dopamine. She was eventually transitioned to oral midodrine. A similar case report highlighted successful minoxidil toxicity management using midodrine after initial vasopressor administration [2]. This was mirrored in the patient’s quick transition to midodrine after phenylephrine use, potentially shortening their ICU stay.

Dose-dependent fluid retention and tachycardia are known side effects of oral minoxidil, sometimes severe enough to limit its use for managing hypertension. Drug manufacturers have even recommended using a diuretic and beta-blocker with minoxidil for treatment-resistant hypertension to counteract fluid retention and tachycardia [1]. These side effects have been well described in the previous studies as well with one study reporting the development of pulmonary edema within 24 hours of minoxidil overdose [3]. In cases of acute overdose, fluid overload may not be solely due to the side effects of minoxidil; aggressive hydration, often the initial treatment for profound hypotension with non-cardiogenic shock, might also contribute. The pulmonary edema resolved rapidly with a few doses of furosemide. It is challenging to argue that aggressive fluid resuscitation should be avoided to prevent such complications, especially when it is a crucial initial measure to treat hypotension and shock from any etiology until the cause of the shock is identified.

Reflex tachycardia from minoxidil can cause myocardial stress, leading to type II myocardial infarction and, in rare instances, acute Type I myocardial ischemia. Previous studies on minoxidil have shown that ST-segment depression and T-wave inversion can be normal findings lasting for up to two weeks after the initial use of oral minoxidil, but persistent EKG changes should raise suspicion for underlying myocardial ischemia. In our case, the patient had EKG changes with elevated troponin suggestive of subendocardial ischemia, both of which normalized rapidly with the resolution of tachycardia. The invasive coronary intervention wasn’t necessary. Recognizing and clinically correlating EKG changes are crucial to avoid unnecessary interventions.

## Conclusions

Minoxidil, a widely used topical hair loss treatment available over the counter, poses severe risks if ingested orally, emphasizing the urgency of managing overdoses. Swift identification and treatment strategies are vital. Management revolves around robust hydration, often coupled with vasopressor assistance for profound circulatory shock. Midodrine, an oral alpha-1 agonist, proves effective, possibly shortening IV vasopressor use and ICU stays.
